# Case report: Infantile generalized pustular psoriasis with IL36RN and CARD14 gene mutations

**DOI:** 10.3389/fgene.2022.1035037

**Published:** 2023-01-10

**Authors:** Xinyun Tong, Yang Li, Xianfa Tang, Yantao Ding, Yao Sun, Liyun Zheng, Yulong Pan, Shengxiu Liu

**Affiliations:** ^1^ Department of Dermatology, First Affiliated Hospital of Anhui Medical University, Hefei, China; ^2^ Key Laboratory of Dermatology (Anhui Medical University), Ministry of Education, Hefei, China; ^3^ Inflammation and Immune-Mediated Diseases Laboratory of Anhui Province, Hefei, China; ^4^ The PLA Navy Anqing Hospital, Anqing, China

**Keywords:** infantile pustular psoriasis, gene mutation, IL36RN, CARD14, AP1S1, biological agents

## Abstract

Infantile pustular psoriasis (IPP) is an extremely rare skin disease associated with genetic factors. Gene mutations of IL36RN (interleukin-36 receptor antagonist), CARD14 (caspase recruitment family member 14), and AP1S1 (the σ1C subunit of the adaptor protein complex 1) had been identified to be involved in the pathogenesis of IPP. IPP usually develops with no preceding psoriasis vulgaris (PV) or familial history. Here, we report a case of a 6-month-old infant and make the diagnosis of IPP by a series of examinations; subsequently, by detecting coexistent mutations of IL36RN and CARD14, the diagnosis is intensified from a genetic point of view. We treated the child with traditional oral and topical drugs regardless of the commonly used acitretin considering its potential side effects, such as skeletal toxicity, and the lesions got conspicuous improvement with much reduction of inflammation. Owing to the genetic mutation of IL-36, there had been reported cases focusing on anti-IL36 biological agents in the treatment of IPP, and it could be a new weapon to treat and improve such IL-36RN-deficient skin diseases.

## Introduction

Generalized pustular psoriasis (GPP) is a unique and rare type of psoriasis, characterized by red patches, desquamation, and sterile pustules, correlated with hyperpyrexia and systemic inflammation. The majority of GPP is usually preceded by or coupled with psoriasis vulgaris (PV), whereas some cases occurred alone without a history of PV. Infantile pustular psoriasis (IPP) is extremely rare and considered to account for 3.5%–16% of childhood psoriasis (Su et al., 2011) and roughly 0.6% of total pustular psoriasis (De Oliveira et al., 2010). The pathogenesis of GPP has not been fully elucidated. The main triggering factors include infections, drugs, pregnancy, and sudden withdrawal of glucocorticoids. Recent research findings have uncovered the key role of genetic mutations (genes IL36RN, CARD14, and AP1S1) in GPP. It is essential for IPP to make early diagnosis and treatment to avoid life-threating complications as superbug infections and sepsis. Herein, we report a case of a 6-month-old infant with IPP who showed coexistence of IL36RN and CARD14 gene mutations.

**FIGURE 1:**
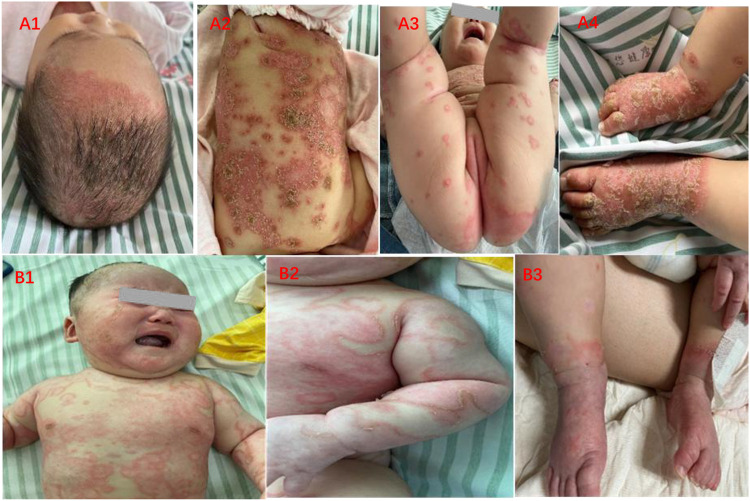
Clinical features of IPP before and after treatment. **(A1–A4)** Widespread erythema, covered thick yellowish scales, and circular distributed pustules on the scalp, trunk, perineum, and extremities. **(B1–B3)** Pink macules with subtle white scales scattered over body, pustules are nearly invisible.

A 6-month-old female child developed scattered rash for half a month and referred to us when erythema and pustules spread widely for 1 week. The initial rash was located on the scalp and hairline, manifesting as papules and pustules, accompanied by diarrhea with watery stools (Figures 1A1–A4). She was referred to a local clinic and was diagnosed to have impetigo herpetifomis, receiving treatment with oral antihistaminic and topical corticosteroid ointment. The scaly erythema and circumjacent pustules integrated into a pus lake and rapidly extended to the entire body. The extremities were covered with thick yellowish-white scales, while nails and oral mucosa were unaffected. The patient developed fever with worsening of her rash for which she was admitted to our inpatient department.

The child was born by natural birth at 38 weeks and had received natural breastfeeding. Through inquiring familial history, her parents and family members have no history of psoriasis or other autoimmune disease. The patient has no hepatosplenomegaly or jaundice with normal chest X-ray. Leucocytes, lymphocytes, monocytes, thrombocytes, and inflammatory indicators such as C-reactive protein and IL-6 were increased. The culture of blood and pustules fluid was negative, indicating the pustules were essentially sterile.

**FIGURE 2:**
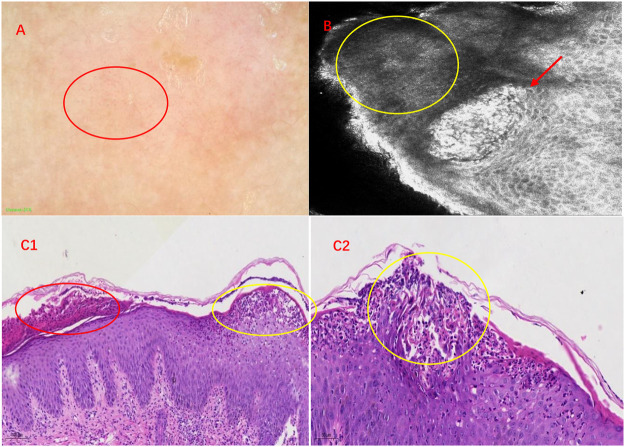
Imaging and histopathological features of IPP. **(A)** Features of IPP under dermoscopy. Under a reddish background, multiple punctate, annular, and hairpin-like vessels are shown by a red circle. **(B)** RCM showed neutrophil infiltrations among keratinocytes in the stratum spinosum, which was called Kogoj’s spongiform pustules (red arrow). The yellow circle displays dyskeratotic cells. **(C1)** Hyperkeratosis, parakeratosis (red circle), acanthosis, formation of subcorneal pustules (Kogoj’s spongiform) (yellow circle), and hemangiectasis with neutrophilic and lymphocyte infiltration in the papillary dermis (HE, ×100). **(C2)** Subcorneal pustules (Kogoj’s spongiform) distributed among acanthocytes (yellow circle) (HE, × 200).

Considering the widespread scaly erythema and pustules, we made the primary diagnosis of GPP. Further examinations to establish the diagnosis were performed, including dermoscopy and reflectance confocal microscopy (RCM). Under the dermoscope, the uniformly distributed punctate, annular, and hairpin-like vessels are shown in a reddish background (Figure 2A). RCM showed hyperkeratosis with parakeratosis, stratum spinosum thickening accompanied by neutrophil infiltrations, and dilation of circuitous vessels in the superficial dermis (Figure 2B). Skin biopsy was performed selecting the lesion with pustules, and histopathological features include hyperkeratosis, parakeratosis, acanthosis, formation of subcorneal pustules, and hemangiectasis with neutrophilic and lymphocyte infiltration in the papillary dermis (Figures 2C1, C2). Among these histopathological features, the most representative and crucial one is subcorneal pustules, which were named as Kogoj’s spongiform. Integrating all the examinations, we finally made the diagnosis of infantile generalized pustular psoriasis.

**FIGURE 3:**
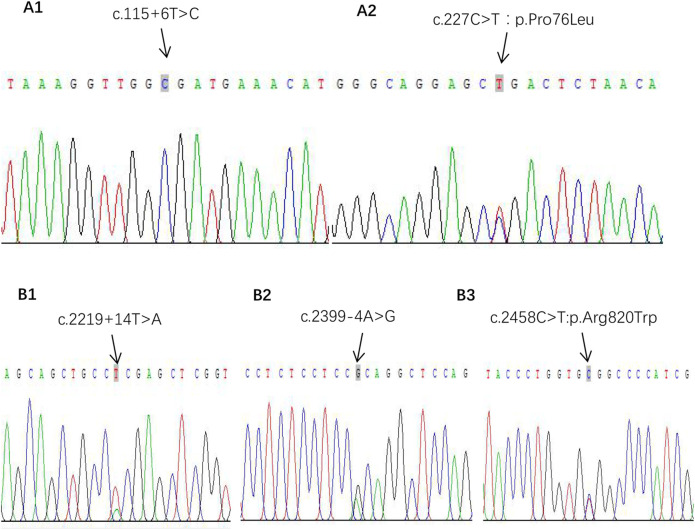
Gene mutations of IPP. **(A1, A2)** Gene IL-36RN pathogenic mutation sites. **(B1–B3)** Gene CARD14 heterozygous mutation sites.

What interests us lies in the pathogenesis of GPP at such a young age of onset since she had no preceding PV or family history of psoriasis. We subsequently detected gene mutations in IL36RN, CARD14, and AP1S1, which were reported closely correlated with the pathogenesis of GPP in literature. It turned out that positive results were discovered in mutations of IL36RN and CARD14, while the results were negative for gene AP1S1. The IL36RN gene exons of E03 showed c.115 + 6T>C homozygous mutation, and c.227C>T:p.Pro76Leu heterozygous mutation on exons of E04 was also detected (Figures 3A1, A2). For gene CARD14, mutation of c.2219 + 14T>A on exon E15 and c.2399-4A>G and c.2458C>T:p.Arg820Trp on exon E18 was analyzed with the heterozygous status (Figures 3B1–B3).

On account of young age and parents’ concerns, we prescribed oral compound glycyrrhizin to regulate immunity, oral antihistamine of chlorpheniramine and desloratadine, topical concussion agent, and ointments such as calcipotriol, desonide, and tacrolimus. After treatment, it could be observed that erythema darkened, scales were decreased, and pustules were gradually absorbed (Figures 1B1–B3).

## Discussion

GPP is an uncommon type of psoriasis first proposed by von Zumbusch in 1910. IPP refers to the onset of GPP within 1 year of age, considered as a rarer form of GPP, and very few cases had been reported. In a study of clinical review of 1,262 childhood psoriasis cases, GPP composed around 0.8% of all young patients, among whom are solely two children less than 2 years of age to suffer from pustular rash (Morris et al., 2001). Unlike adult patients, the most frequent initial affected area is the diaper region among children under 2 years of age. The sudden rash of erythema and sterile pustules disseminate widespread, and pustules may be ring shaped along the margins of erythema, merging into a pus lake in local. Systemic symptoms including fever and fatigue usually occurred as well as abnormal laboratory indicators containing elevated leukocytosis, neutrophilia, and inflammatory cytokines in IPP. Histologically, it typically presents with intraepidermal infiltration of neutrophilia, which was named Kogoj’s spongiform pustules.

The child in our report initially developed erythema and little pustules on the head and face, gradually extended to the trunk, extremities, and diaper region, with fever and elevation of inflammatory markers. Owing to the extremely low morbidity of IPP and no personal or familial history of PV, we primarily made a presumptive diagnosis of acute generalized exanthematous pustulosis (AGEP) or staphylococcal scalded skin syndrome (SSSS). However, no specific medication history and negative bacterial culture are in accordance with the aforementioned diagnosis. In order to clarify the diagnosis, we performed dermoscopy and RCM which tend to take GPP into account. Later, by completing skin biopsy, Kogoj’s spongiform pustules showed by histopathology helped us make the final diagnosis of IPP.

The etiology of GPP remains unclear, and possible factors may be involved: infection, trauma, emotion, drugs, and sudden withdrawal of glucocorticoids (Salem et al., 2009). However, in some cases, especially for children without preceding PV history, the aforementioned factors may be the motivator; the fundamental factor might be associated with gene codes. In some familial case reports, researchers proposed that GPP was connected with genetic factors (Hubler, 1984). In subsequent studies, gene mutations of IL36RN, CARD14, and AP1S1 were identified to be involved in pathogenesis. Considering the child in our case had neither personal nor familial history of psoriasis and early onset of uncommon IPP, we highly suspect there may be genetic mutations contributing to IPP onset.

Interleukin-36 cytokines include IL-36α, IL-36β, IL-36γ, and IL-36 receptor antagonist (IL-36Ra) that belong to the interleukin-1 family. IL-36s is expressed in various cell types, including keratinocytes and immune cells (Bassoy et al., 2018). The combination of IL-36s and IL-36 receptor (IL-36R) could activate nuclear factor-κB (NF-κB) and mitogen-activated protein kinase (MAPK) pathways to promote inflammatory waterfall. IL-36Ra reacts as a regulator by binding to IL-36R in a competitive manner, so as to impair the inflammatory effects of IL-36s (Hussain et al., 2015). IL36RN encodes IL-36Ra; the mutation of IL-36RN inhibits the function of IL-36Ra, leading to excessive activation of IL-36-associated signal pathways, and, thus, exacerbates GPP inflammations (Marrakchi et al., 2011). In acute GPP, the phenomenon that IL36RN mutations result in reduced IL36Ra is named DITRA (deficiency of IL-36Ra), and DITRA has been described as an independent and innate immunity-mediated autoinflammatory disease.

In 2011, Marrakchi et al. (2011) reported homozygous mutations of IL36RN in Tunisian families, indicating that familial GPP (FGPP) is inclined to be autosomal recessive inherited diseases. Later on, mutations of IL36RN in sporadic GPP were detected successively. Mutations in IL36RN have been reported to occur in 23%–37% patients with GPP (Körber et al., 2013). So far, no less than 20 IL36RN gene mutations had been found to be associated with the pathogenesis of GPP, among which c.115 + 6T > C has the highest mutation rate in Asian populations. Li et al. (2014) investigated GPP in the Chinese Han population; they discovered that the frequency of the IL36RN mutation in pediatric-onset GPP was higher than that in adult-onset GPP, suggesting that IL36RN genetic alterations possibly play a major role in the onset of GPP for an early age. Hussain et al. (2015) analyzed genotypes of IL36RN in 233 GPP patients and concluded that patients carrying the IL36RN mutation had a younger age of onset, a lower probability of PV before or during the onset of GPP, and were easier to suffer systemic inflammation. GPP is etiologically independent of PV, on the basis of the mutation of IL36RN in GPP alone compared to GPP with PV cases, indicating GPP is a unique sub-type of psoriasis (Sugiura et al., 2013).

Our research detected the IL36RN gene exon sequence showing c.115 + 6T>C homozygous mutation and c.227C>T:p.Pro76Leu heterozygous mutation in a 6-month-old infant, in accordance with previous research. Wang et al. (2017) put forward that the mutation of c.227C>T tends to be accompanied by the c.115 + 6T>C mutation, indicating some mutation sites may show negative regulatory effects to avoid threatening inflammations.

Other genes associated with GPP include CARD14 (caspase recruitment domain-containing protein 14) and AP1S3. CARD14 is located within PSORS2 and encodes the nuclear factor (NF)-kB activator. CARD14 mutations mainly occurred in psoriasis vulgaris (PV) according to previous studies (Jordan et al., 2012). The incidence of CARD14 heterozygous mutations in GPP with PV is up to 21% (Neuhauser et al., 2020). Li et al. (2019) set forth that the CARD14 mutation lies in PV and GPP with PV, while being negative in GPP alone. Qin et al. (2014) elaborated that CARD14 was significantly correlated with GPP, but no obvious differential was observed when comparing with the PV group.

After detecting mutations of IL36RN, we further detected gene CARD14, and it turned out that mutations of c.2219 + 14T>A on exon E15 and c.2399-4A>G and c.2458C>T:p.Arg820Trp on exon E18 were all in the heterozygous status. Among these mutations, CARD14 p. Arg820Trp (rs11652075) was found to be a PV-susceptible variant in a large psoriasis cohort (Jordan et al., 2012). Our child belongs to IPP alone; it seems the result of the negative pathogenic mutation in CARD14 is consistent with Li’s perspective. What differs from their results lies in the benign mutations of this gene, refreshing the cognition of the relationship between the CARD14 genotype and the clinical phenotype of GPP. Further research is needed to know whether such benign mutations have an effect on GPP to verify the exact pathogenic mechanism for the CARD14 mutation in GPP and even IPP.

Studies have verified that the impetigo mediated by both mutations of IL36RN and CARD14 are included in a group of autoinflammatory keratoderma (Akiyama et al., 2018). The pathogenicity of IL-36RN and CARD14 mutations both involve NF-κB and downstream MAPK signaling pathways, which may explain their interaction owing to mutual inflammatory pathways. We believe the coexistence of IL-36RN and CARD14 mutations, although benign mutation, may act together to induce GPP and promote the early age of onset, but further investigations are needed to elucidate this hypothesis and associated signal pathway mechanisms.

The AP1S3 mutation is mainly observed in Europeans, barely in Asians, which may coexist with the IL36RN mutation and alter the phenotypic effects of the latter. We also detected AP1S1, and the result turned out to be negative, which is consistent with the former conclusions, manifesting the virulence genes may diverse in different races.

To date, there has been no GPP-specific therapeutic guidance due to low prevalence and lack of randomized controlled trial statistics. Traditional therapies include acitretin, ciclosporin, methotrexate, dapsone, topical ointment, and PUVA phototherapy. Biologics have been approved for treatment of GPP, and multiple clinical research studies have verified the effectiveness and safety of biologics applied in treatment for GPP. The applications of biologics including TNFα inhibitors (Infliximab), antagonists of IL-17 (secukinumab, ixekizumab, and brodalumab), and IL-23 (guselkumab) had been proved to improve prognosis and reduce complications.

With the elaboration of the immune-inflammatory mechanism for GPP, researchers later focused on therapies that target cytokines of IL36 and IL-1β, on the basis of their abnormal activations in GPP compared with PV. Case reports of agents targeting IL-1 (recombinant IL-1 receptor antagonist anakinra) showed it effective for GPP (Huffmeier et al., 2014). Several clinical research studies attempted to apply agents targeting IL-36 or IL-36R. In a trial of novel anti-IL-36R biologics treated with seven GPP patients who were given single-dose spesolimab (anti IL-36R) intravenously, their lesions alleviated rapidly (Bachelez et al., 2019). However, the mutation of IL36RN does not influence the efficacy of biologics applied in GPP in view of reported research studies (Kromer et al., 2021).

For IPP, systemic application of acitretin had been proven to be safe and effective (Ergin et al., 2008). However, the side effects of acitretin including skeletal toxicity, although with low incidence, may result in hypogenesis, so it should be discreet to select acitretin, especially for children in the critical growth period. Children with GPP, with or without the IL36RN mutation, responded well to oral low-dose acitretin, but IL36RN-positive cases suffered a much higher half-year recurrence rate after the withdrawl of acitretin treatment. The child in our case was 6 months old and had no systemic complications such as bacterial infections. By weighing the pros and cons, we prescribed compound glycyrrhizin to regulate immunity, cephalosporin antibiotics owing to elevated inflammatory factors, antihistamine, and topical treatment, including calcipotriol, tacrolimus, and glucocorticoid ointment. During the treatment, the pustules were absorbed accompanied by erythema which faded away. The temperature kept normal, and inflammatory indicators improved after reexamination. Therefore, systemic anti-inflammatory and immune-regulatory therapy in combination with topical therapy is effective even without systemic administration of acitretin. During the follow-up after discharge, the child visited our outpatient department regularly, we continued to treat her with oral and topical drugs as inpatient prescriptions, and she did not recur with pustules, only scattered erythema occurred.

In summary, we verified the gene mutations of IL36RN and CARD14 in a 6-month-old infant diagnosed as IPP. On the one hand, physicians could recognize this rare disease at an earlier stage, especially for young children without personal or family history of PV, so as to improve the prognosis. On the other hand, patients with gene mutations have opportunities to receive biological agents, such as novel targeting IL-1 or IL-36 monoclonal antibody. Randomized controlled clinical studies with large samples are still needed to verify the clinical efficacy of these biologics in patients with GPP and IPP.

## Materials and methods

This study was performed in adherence with the principles of the Declaration of Helsinki and was approved by the Ethical Review Committee of Anhui Medical University.

Clinical images were taken using a digital camera. Punch biopsy specimens were obtained from upper limb skin lesions. Paraffin sections were prepared, stained with hematoxylin and eosin, and examined by light microscopy. Examinations were performed using both dermoscopy and RCM. All dermoscopic (Dermoscopy-II; Dermat^®^, Beijing, China) and RCM (Vivascope 3,000^®^; Lucid Inc. Rochester, NY, United States) images were captured appropriately and analyzed.

The kits of gene detection mainly include the TIANamp Blood DNA Kit, Premix Taq (Ex Taq Version 2.0), BigDye™ Terminator v3.1 Cycle, Sequencing Kit, and BigDye XTerminator Purification Kit. The results were analyzed by using a 3730XL sequenator to perform Sanger sequencing.

## Data Availability

The datasets for this article are not publicly available due to concerns regarding participant/patient anonymity. Requests to access the datasets should be directed to the corresponding author.
